# Integrating Western Medicine With Japanese Traditional Medicine for Primary Health Care During Natural Disasters and Pandemics: Lessons From Japan

**DOI:** 10.7759/cureus.90323

**Published:** 2025-08-17

**Authors:** Shin Takayama

**Affiliations:** 1 Department of Education and Support for Regional Medicine, Tohoku University Hospital, Sendai, JPN

**Keywords:** acupuncture treatment, covid-19 pandemic, disaster exercise, kampo medicine (japanese herbal medicine), public health problems

## Abstract

Natural disasters and pandemics create major challenges for public health systems, requiring efficient medical responses under constrained conditions. Ensuring comprehensive healthcare in disaster-stricken areas is critical, as conventional medical resources may be insufficient in some settings. Japanese traditional (Kampo) medicine (JTM) offers a potential solution for enhancing healthcare delivery in these scenarios. This work was motivated by the desire to facilitate the international dissemination of the integration of Western medicine and traditional medicine.

Japan has integrated JTM, including herbal medicine, acupuncture, and massage therapy, with conventional medical care in disaster and pandemic responses. JTM has been applied in disaster settings to help manage a wide range of conditions, including respiratory infections, hypothermia, gastrointestinal disturbances, allergic reactions, psychiatric symptoms, and musculoskeletal pain.

Japan’s unique medical system, where physicians are trained in both Western and JTM, allows for their seamless integration in healthcare and disaster relief efforts. Furthermore, herbal medicines are covered by the National Health Insurance System.

More recently, JTM has also been integrated with conventional treatments during infectious disease outbreaks. Early administration appeared beneficial in preventing disease progression and in alleviating symptoms, particularly among individuals without prior immunization.

The successful integration of JTM in disaster and pandemic management highlights its role as a complementary healthcare approach. Japan’s model demonstrates the potential benefits of combining traditional and Western medicine and provides valuable insights for other countries seeking to enhance disaster preparedness and healthcare resilience.

## Editorial

Introduction　

Natural disasters and pandemics disrupt public health systems and require effective medical responses under constrained conditions. Disasters, such as earthquakes, tsunamis, and floods, destroy infrastructure, forcing evacuees to live in temporary shelters with poor sanitary conditions. The Great East Japan Earthquake (magnitude: 9.0), with a subsequent tsunami, inflicted immense damage over a wide area of eastern Japan on March 11, 2011. Railways, roads, cars, houses, and infrastructure on the east coast of Japan were destroyed [1].

Pandemics, such as coronavirus disease 2019 (COVID-19), require efficient infection control and treatment strategies despite limited resources. Therefore, traditional medicine, particularly traditional Japanese traditional (Kampo) medicine (JTM), was integrated with conventional medicine in Japan to address health challenges during these crises. Understanding how JTM has been utilized in disasters and pandemic scenarios provides insights into its role in comprehensive healthcare strategies.

This work was motivated by the desire to facilitate the international dissemination of the integration of Western medicine and traditional medicine.

Approach

Japan has adopted an integrative approach by incorporating JTM into its disaster and pandemic medical responses. This includes the use of herbal medicines, acupuncture, and massage therapy, along with conventional treatments. Since Kampo examinations can be performed without the use of medical equipment, they are easier to use in such scenarios. Following the 2011 Great East Japan Earthquake, evacuees suffered from common cold, hypothermia, enterocolitis, allergic reactions, psychiatric symptoms, and musculoskeletal pain, due to harsh living conditions [1]. Although the diagnosis was limited in these situations, the symptoms were alleviated through integrative medicine. Recorded data contributed to personal identification, safety support, and confirmation of symptom relief progress, as well as research. Further data collection and evidence will be needed when using this as a tool for establishing policies on the integration of Western medicine and traditional Japanese medicine. Physicians worked with acupuncturists and massage therapists in evacuation centers to establish a collaborative model of care. Acupuncture and massage are particularly effective for managing pain, stress, and insomnia, with evacuees reporting high satisfaction rates. Medical records are used in the support operation. Recorded data contributed to personal identification, safety support, confirmation of the progress of symptom relief, and also to research. Similar applications were observed in subsequent disasters, such as the 2015 Joso City flood, the 2016 Kumamoto earthquake, and the 2024 Noto Peninsula earthquake [2]. An observational study showed that acupuncture and massage therapy significantly decreased the median Face Scale score of subjective symptoms in evacuees (P < .001) [3].

Table 1 shows the list of JTM formulas used for health management during evacuation after disasters, according to past experience and activity reports. The table includes information on personalized Kampo treatment and randomized controlled trials for post-traumatic stress disorder (PTSD). The efficacy of JTM Saikokeishikankyoto (SKK) in treating PTSD following the Great East Japan earthquake and tsunami was reported in a randomized controlled trial (RCT). SKK significantly improved the Revised Impact of Event Scale score (P < 0.001), particularly reducing avoidance (P = 0.003), hyperarousal (P < 0.001, and intrusion (P < 0.001) [4]. The efficacy of the traditional Chinese medicine Xiao-Tan-Jie-Yu-Fang (XTJYF) in treating the psychological state of Great Sichuan earthquake survivors with PTSD was reported in a double-blind RCT. XTJYF significantly improved the Revised Symptom Checklist-90 index, particularly with regard to somatisation, obsessive-compulsive behaviour, depression, anxiety, hostility, and sleep deprivation [5].

**Table 1:** The list of JTM formulas for the management of healthcare during evacuation after disasters. This table is modified from [5]. JTM: Japanese traditional (Kampo) medicine

Disease, symptom, and condition	JTM formulas
Post-traumatic stress disorder	Saikokeishikankyoto (Chai-Hu-Gui-Zhi-Gan-Jiang-Tang)
Common cold	Kakkonto (Ge-Gen-Tang)
	Maobushisaishinto (Ma-Huang-Fu-Zi-Xi-Xin-Tang)
Sore throat	Kikyoto (Jie-Geng-Tang)
Hypothermia	Tokishigyakukagoshuyushokyoto (Dang-Qui-Si-Ni-Jia-Wu-Zhu-Yu-Sheng-Jiang-Tang)
Enterocolitis	Rikkunshito (Liu-Jun-Zi-Tang)
	Goreisan (Wu-Ling-San)
Rhinitis	Shoseiryuto (Xiao-Qing-Long-Tang)
Dry cough	Bakumondoto (Mai-Men-Dong-Tang)
Constipation	Mashiningan (Ma-Zi-Ren-Wan)
Insomnia	Sansoninto (Suan-Zao-Ren-Tang)
Irritation	Yokukansan (Yi-Gan-San)
Anxiety	Kamikihito (Jia-Wei-Qui-Pi-Tang)
Fatigue	Hochuekkito (Bu-Zhong-Yi-Qi-Tang
Abdominal pain (abdominal fullness)	Daikenchuto (Da-Jian-Zhong-Tang)
Leg edema or numbness of lower extremities	Goshajinkigan (Niu-Che-Shen-Qi-Wan)
Cramping in the calves	Shakuyakukanzoto (Shao-Yao-Gan-Cao-Tang)
Dizziness or vertigo	Hangekobokuto (Ban-Xia-Hou-Po-Tang)
	Hangebyakujutsutemmato (Ban-Xia-Bai-Zhu-Tian-Ma-Tang)
	Ryokeijutsukanto (Ling-Qui-Zhu-Gan-Tang)
	Orengedokuto (Huang-Lian-Jie-Du-Tang)
Chest pain due to post-traumatic stress disorder	Daisaikoto (Da-Chai-Hu-Tang)
Tongue pain	Yokukansan (Yi-Gan-San)
Psychosomatic symptoms (Anxiety, low-grade fever, fatigue, coldness, sweating, dry mouth, shoulder stiffness, and dizziness）	Saikokaryukotsuboreito (Chai-Hu-Jia-Long-Gu-Mu-Li-Tang)

During the COVID-19 pandemic, JTM was incorporated into treatment plans in clinical settings and research. The Integrative Management in Japan for Epidemic Disease (IMJEDI) project classifies its use into prevention, treatment, and recovery stages. Historical experience with the Spanish flu and findings of recent clinical studies have demonstrated the potential of JTM in reducing symptom severity and preventing disease progression, especially in non-vaccinated patients.

Local setting

The Japanese healthcare system uniquely supports the integration of Kampo and Western medicine. Physicians are trained in both and can prescribe JTM under the National Health Insurance System. Since 2001, medical education in Japan has included JTM in its core curriculum, ensuring that all licensed doctors are familiar with its applications.

Organizations such as the Japan Medical Association and Japan Liaison Council for Disaster Acupuncture and Massage (JLCDAM, https://jlcdam.net/) have promoted the use of acupuncture and massage therapy in disaster scenarios. The JLCDAM collects clinical data to evaluate the effectiveness of traditional medicine in disaster responses, in collaboration with several acupuncture and moxibustion organizations, contributing to evidence-based integration. The Japanese Society for Oriental Medicine also provides online resources for physicians detailing appropriate Kampo treatments for disaster-related health issues. The Japan Medical Association Team also prepares JTM formulas as medicines for use in disaster-stricken areas.

Relevant changes

On March 11, 2020, the World Health Organization (WHO) declared COVID-19 a global pandemic based on the spread and severity of the infection [6]. Integration of JTM evolved through systematic research and real-world applications. During the COVID-19 pandemic, large-scale studies were conducted to assess the efficacy of JTM. A nationwide observational study compared patients with COVID-19 who received Kampo and conventional treatments, with those receiving conventional treatments alone. This research revealed that early Kampo interventions reduced the risk of illness worsening, particularly in patients who had not received steroids. The efficacy of the combined use of kakkonto and shosaikotokakikyosekko added to conventional treatment in patients with COVID-19 was reported in an RCT. The result showed that fever (hazard ratio [HR] 1.68, 95% confidence interval [CI] 1.00-2.83, p = 0.0498) and shortness of breath (HR 2.15, 95% CI 1.17-3.96, p = 0.0141) disappeared faster with the combined use of kakkonto and shosaikotokakikyosekko in unvaccinated cases [7]. These findings reinforce the value of JTM as a complement to modern antiviral treatments. Moreover, JTM’s affordability and long history of safety make it a viable option in resource-limited disaster situations. Kampo medicine extract formulations are easier to distribute and administer than traditional decoctions, increasing their practicality in emergency settings.

Traditional medicine has a long history of application in many cases of pandemics over the past thousand years and has served as complementary medicine in periods before the development of new drugs and vaccines. It is also inexpensive from a medical economic perspective. During the pandemic, Japanese physicians treated mild to moderate COVID-19 patients in isolation facilities using conventional drugs and JTM formulas. Infection control measure was successfully performed in collaboration with public health experts, infection control doctors, nurses, and pharmacists. Moreover, operations and support were recorded in the same medical record systems in the respective core hospitals. In Japan, medical staff, including physicians, nurses, and pharmacists, use a uniform medical record and were trained in JTM as students. This system was successful in facilitating collaboration during the pandemic, ensuring that valuable medical information was garnered for research and public health services.

Lessons learnt

Japan’s experience highlights the benefits of integrating traditional and Western medicine in disaster and pandemic responses, with key takeaways including a holistic care approach. JTM addresses both physical and psychological symptoms, providing comprehensive care beyond conventional treatments; collaborative medical teams, where the integration of acupuncturists, massage therapists, and physicians in disaster relief efforts enhances healthcare delivery; data collection and research, through which systematic documentation of JTM’s effects in disaster scenarios strengthens its credibility and facilitates evidence-based practice; education and policy support, ensuring that the inclusion of JTM in medical training allows future physicians to utilize it effectively in crisis situations; and economic and practical advantages, as JTM’s cost-effectiveness and ease of distribution make it a valuable resource in disaster relief. Japan’s integrative approach serves as a model for other countries seeking to enhance disaster preparedness and healthcare resilience, demonstrating that by incorporating traditional medicine along with modern treatments, healthcare systems can optimize patient outcomes during emergencies.

Conclusion

JTM addresses physical and psychological symptoms and provides comprehensive care beyond conventional treatments. Systematic documentation of the effects of JTM in disaster scenarios strengthens its credibility and facilitates evidence-based practice. Including JTM in medical training ensures that future physicians can utilize it effectively in crisis situations.
